# Beyond Bartonellosis: A Case Report of Hepatic Tuberculosis as a Final Diagnosis in a Child With Down Syndrome

**DOI:** 10.1155/crdi/3276122

**Published:** 2026-09-07

**Authors:** Paula Camargos, Antonio Oliveira Filho, Joaquim Bustorff Silva, Márcia Cavalaro da Silva, Maria Giovana Farias, Priscilla Tosta, Ricardo Pereira, Mariana Tresoldi das Neves Romaneli

**Affiliations:** ^1^ Hospital de Clínicas, School of Medical Sciences, University of Campinas (UNICAMP), Campinas, São Paulo, Brazil, unicamp.br; ^2^ Department of Pediatric Surgery, School of Medical Sciences, University of Campinas (UNICAMP), Campinas, São Paulo, Brazil, unicamp.br

**Keywords:** *Bartonella* infections (or bartonellosis), case report, child, hepatic, tuberculosis

## Abstract

Hepatic tuberculosis is a rare form of extrapulmonary manifestation. This case report depicts a 6‐year‐old boy with Down syndrome who presented with prolonged fever, weight loss, and hepatomegaly. The investigation revealed liver abnormalities suggestive of a granulomatous process, with the initial diagnosis of bartonellosis and, later, tuberculosis, with molecular testing and culture from a fragment of the liver biopsy. He underwent treatment and exhibited an excellent clinical response. This case highlights the importance of including hepatic tuberculosis in the differential diagnosis of liver diseases in children, even in the absence of evident pulmonary signs, especially in endemic regions including Brazil.

## 1. Introduction

Tuberculosis is endemic in Brazil, with 84,308 new cases in 2024, of which 4.1% occurred in children and adolescents up to 15 years of age. In this age group, the proportion of extrapulmonary forms (20.6%) is higher than that in the general population [1, 2]. The disease can affect practically all organs, even in the absence of pulmonary involvement. In the abdominal form, the liver can be reached by hematogenic or lymphatic dissemination, leading to the formation of hepatic granulomas with central caseous necrosis, surrounded by epithelioid cells, Langhans giant cells, and lymphocytes—a characteristic histopathological pattern [3, 4]. The symptoms include fever, adynamia, inappetence, weight loss, hepatomegaly, ascites, splenomegaly, and abdominal pain. Laboratory tests reveal increased evidence of inflammatory activity, altered liver enzymes, leukocytosis, anemia, and hypoalbuminemia; elevated alkaline phosphatase suggests hepatic infiltration [4]. Imaging findings include hypoechoic lesions and complex masses on ultrasound, hypodense tuberculomas with peripheral enhancement on tomography, and hepatic calcifications. Regional lymph nodes with caseous necrosis exhibit central hypoattenuation with peripheral enhancement [4, 5].

Noteworthy among the differential diagnoses is bartonellosis caused by *Bartonella henselae*, transmitted by scratching or cat bite. It can occur with localized or systemic infection, involving the liver, spleen, central nervous system, bones, eyes, skin, and heart [6]. In this case, the main symptoms are prolonged fever, abdominal pain, weight loss, headache, arthralgia, and chills. Hepatosplenomegaly occurs in up to 50% of the cases, while lymphadenopathy is absent in more than half, making diagnosis difficult. Transaminases usually remain normal. Although several drugs have in vitro activity against *Bartonella*, such as macrolides, fluoroquinolones, and doxycycline, only gentamicin and rifampicin are recommended for systemic cases, with treatment durations ranging from 5 days to 6 weeks, depending on the severity and immune status of the patient [6].

The case presented is of a child with Down syndrome who presented with liver damage due to *Bartonella spp.* and *Mycobacterium tuberculosis*, which justifies the rarity of the case.

## 2. Case Report

A 6‐year‐old boy with Down syndrome, asthma, and hypothyroidism was admitted to a tertiary hospital with daily fever, weight loss of 5 kg in 1 month, and a palpable abdominal mass in liver topography. Ultrasound revealed a mass in the left lobe and splenomegaly. A CT scan of the abdomen showed a hypodense 8 × 5‐cm lesion, two smaller nodules with calcifications in the hepatic V segment, and regional lymph nodes with calcifications.

Referred to a reference oncological center, he underwent biopsy of the mass and mesenteric lymph node, whose histological study showed extensive coagulative necrosis with fibrosis, focal lymphohistiocytic infiltrate, and Langhans cells, without identification of fungi or alcohol–acid resistant bacilli. Excluding oncohematological disease, he returned to the tertiary hospital, where laboratory tests showed anemia, elevated inflammatory tests, and increased alkaline phosphatase. Serologies for HIV, toxoplasmosis, mononucleosis, cytomegalovirus, and paracoccidioidomycosis were negative. An initial tuberculosis screening was also performed prior to target therapy, including negative chest imaging (radiography and CT), a nonreactive tuberculin skin test (TST), and a negative epidemiological investigation of the parents; an interferon‐gamma release assay (IGRA) was unavailable. Additionally, given the patient’s increased susceptibility to infections, a basic immunological assessment, including a complete blood count, serum immunoglobulins (IgG, IgA, IgM, and IgE), complement fractions (C3 and C4), HIV serology, and vaccine‐induced humoral immunity (hepatitis B and rubella), revealed no significant abnormalities. Genus‐specific PCR for *Bartonella henselae* was positive, and treatment with azithromycin was instituted for 6 weeks, resulting in slight clinical improvement. The only finding supporting the diagnosis of *Bartonella* infection was the positive PCR result obtained from peripheral blood. No clinical features specifically suggestive of cat‐scratch disease were identified.

**FIGURE 1:**
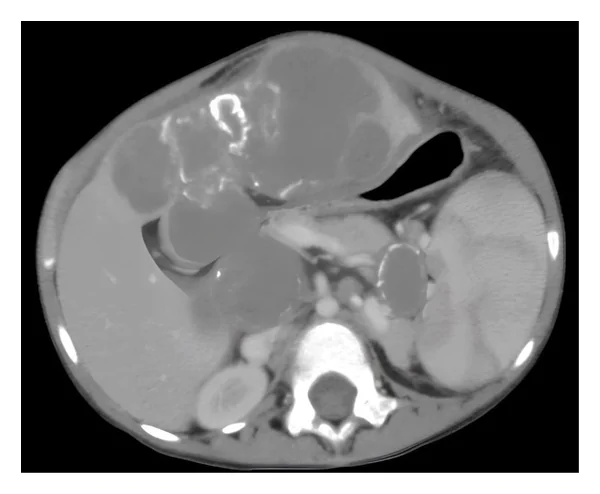
Abdominal tomography with contrast in portal phase, axial section showing heterogeneous liver mass in the left lobe (9.7 × 5.6 × 11.8 cm), with coarse calcifications. There are also two other solid lesions with characteristics similar to the aforementioned, one in the caudate lobe (7.4 × 3.3 × 4.0 cm) and the other in segment VI, measuring (2.5 × 2.7 × 2.1 cm). Lymph node in left hypochondrium with necrotic center and peripheral calcification. Splenomegaly and free fluid are observed in the abdominal cavity.

**FIGURE 2:**
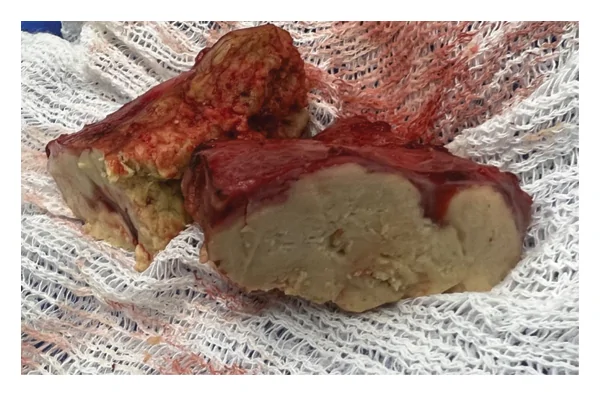
Surgical piece. Two liver fragments measuring 5.5 × 3.5 cm and 5.2 × 5.5 × 2.3 cm and weighing 75.0 g, partially covered by brownish and fibrous Glisson’s capsule. The fragments present infiltrative and whitish lesions measuring 5.0 × 5.0 × 2.3 cm and 4.5 × 3.5 cm, respectively, of firm‐elastic consistency, presenting an irregular partially smooth and evenly bloody surface. At the cuts, lesions have a smooth and homogeneous whitish surface, with fibroelastic tissue in between.

After 1 year of outpatient follow‐up, there was an increase in liver mass (Figure 1) and the appearance of new lesions. Partial hepatectomy was, then, indicated. Molecular rapid test and surgical specimen culture (Figure 2) were positive for *Mycobacterium tuberculosis* although AFB smear remained negative.

**FIGURE 3:**
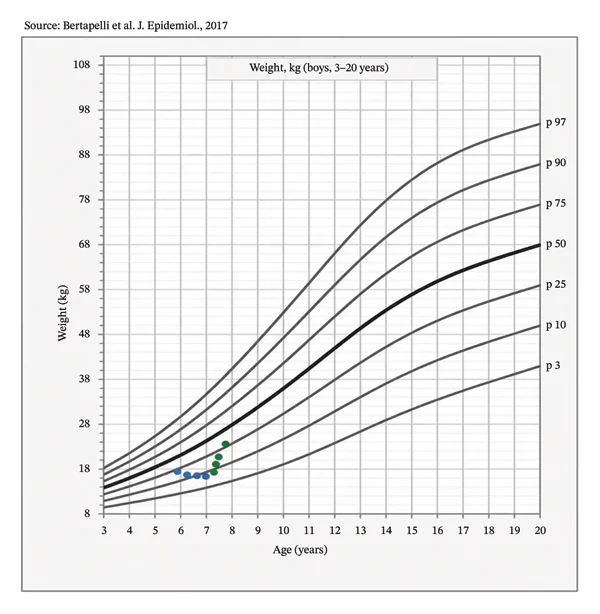
Patient weight‐for‐age growth chart. Growth curves for boys aged 3–20 years based on data from Bertapelli et al. [7]. Blue plot points indicate weight progression prior to antituberculosis treatment; green plot points depict posttreatment catchup growth.

Antitubercular treatment was initiated in accordance with the Brazilian Ministry of Health guidelines for extrapulmonary tuberculosis: an intensive phase with isoniazid, rifampicin, and pyrazinamide (RHZ) for 2 months, followed by a maintenance phase with isoniazid and rifampicin (RH) for 4 months, totaling 6 months of therapy [1]. The patient showed good tolerance, with no hepatotoxicity, and a favorable clinical course, featuring resolution of abdominal pain, weight recovery, and normalization of laboratory test results (Table 1). No follow‐up imaging studies were performed. Prior to intervention, the patient demonstrated poor weight gain, remaining below expected growth percentiles (blue points, Figure 3). Following the initiation of antituberculosis therapy, significant catchup growth was observed, returning the patient toward his baseline growth percentile curve (green points, Figure 3). No index case was identified during the contact investigation of household family members. Following diagnosis and treatment initiation, the patient was monitored on an outpatient basis and was formally discharged from the pediatric infectious disease service 1 year after completing tuberculosis treatment.

**TABLE 1:** The evolution of laboratory test results.

Exam	Prebiopsy	Posttreatment for bartonellosis	Start of treatment for tuberculosis	Reference range
Hb (g/dL)	9.3	9.5	13.1	M: 14–18/W: 12–16
Ht (%)	29.9	30.1	40.2	M: 41–52/W: 36–46
MCV (fL)	69.5	66.4	74.3	80–99
Leukocytes (× 10^3^/μL)	9.50	8.63	6.00	4.0–10.0
Platelets (× 10^3^/μL)	389	314	314	150–400
CRP (mg/L)	119	115	53	Values above 100 suggest bacterial infection
Albumin (g/dL)	—	2.9	3.8	3.5–5.2
AST (U/L)	38	67	24	15–60
ALT (U/L)	27	48	18	13–45
GGT (U/L)	32	70	53	3–22
FAL (U/L)	220	460	251	124–335
Bilirubins (mg/dL)	0.38		0.33	0.3–1.2

*Note:* Hb = hemoglobin; Ht = hematocrit; ESR = sedimentation rate; AST = aspartate aminotransferase; ALT = alanine aminotransferase; GGT = glutamyltransferase range; ALP = alkaline phosphatase; M = men; W = women.

Abbreviations: CRP, C‐reactive protein; MCV, mean corpuscular volume.

## 3. Discussion

The developing child’s immune system is naturally more vulnerable, especially in the first years of life, and in patients with trisomy 21, additional changes increase susceptibility to infections. Children with Down syndrome often exhibit abnormalities, even in the presence of normal basic immunological screening tests, such as elevated IgE, reduced IgM, lymphopenia, increased CD8+ lymphocytes, decreased CD4+ lymphocytes, and disturbances in chemotaxis, which predispose them to viral, bacterial, and fungal infections [8, 9].

Positivity for *Bartonella henselae* initially directed the hypothesis to systemic bartonellosis. The symptoms are nonspecific and coinfections with other agents may occur in immunosuppressed patients. The diagnosis of bartonelose is complex, and there is no gold‐standard methodology. The combination of liquid and solid media culture, multiple PCR tests, and serology is used to increase sensitivity as in the present case [10, 11].

National studies show the circulation of *Bartonella spp.* in asymptomatic individuals. Pitassi et al. identified DNA from *Bartonella spp.* in 3.2% of 500 blood donors, a rate that increased to 23% when the same samples were analyzed with four distinct PCR methodologies. It is believed that *Bartonella spp.* is able to modulate the host immune system by stimulating the secretion of IL‐10, which inhibits the response of T helper cells, monocytes/macrophages, and dendritic cells. This mechanism may favor the persistence or reactivation of opportunistic agents, such as *Mycobacterium tuberculosis* [10–12].

During treatment for bartonelose, the patient only showed partial improvement. Considering the progression of liver injury and lymph node enlargement, and considering that the first biopsy had been inconclusive, a more invasive surgical approach was chosen, which allowed the definitive diagnosis. In solid nodular lesions, the confirmation of tuberculosis depends on culture or rapid molecular test since bacilloscopy tends to be negative in the absence of liquefied material, as happened in this case [5].

## 4. Conclusion

Hepatic tuberculosis is rare, usually associated with involvement of other abdominal organs and regional lymph nodes. Symptoms are nonspecific and diagnosis represents a clinical challenge, especially in children with immunological changes. In endemic areas, investigation should be persistent, even in the absence of index case and when initial treatment, based on another identified agent, does not lead to significant improvement. The prognosis tends to be favorable when the diagnosis is established and treatment instituted early.

## Funding

This study received no funding.

## Ethics Statement

The study was reviewed and approved by the Research Ethics Committee following ethical guidelines.

## Consent

Written informed consent was obtained from the patient and parent for publication of this case report and accompanying images.

## Conflicts of Interest

The authors declare no conflicts of interest.

## Supporting Information

Additional supporting information can be found online in the Supporting Information section.

## Supporting information


**Supporting Information** This case report has been prepared in accordance with the CARE (CAse REport) guidelines, and the CAREchecklist is provided as a supporting information (CAREchecklist.pdf).

## Data Availability

Data sharing is not applicable to this article as no datasets were generated or analyzed during the current study.
